# Effects of small ridge and furrow mulching degradable film on dry direct seeded rice

**DOI:** 10.1038/s41598-020-79227-9

**Published:** 2021-01-11

**Authors:** Hui Li, Shan Zeng, Xiwen Luo, Longyu Fang, Zhanhao Liang, Wenwu Yang

**Affiliations:** 1 College of Biological and Agricultural Engineering, Jilin University, Changchun, 130022 China; 2 Key Laboratory of Key Technology on Agricultural Machine and Equipment, Ministry of Education, South China Agricultural University, Guangzhou, 510642 China

**Keywords:** Ecology, Environmental sciences

## Abstract

Global climate change and socio-economic development have led to a shortage of water and labour resources, which has had a significant impact on rice cultivation. In this study, the application of micro-ridge-furrow planting technology and degradable film mulching in dry direct-seeded rice was investigated to address the factors restricting the development of the rice industry and reduce the impact of rice production on the environment. The effects of a micro-ridge-furrow planting pattern and degradable film mulching on soil temperature, seedling growth, and yield of dry direct-seeded rice in a semiarid region of China were studied through three field experiments: micro-ridge-furrow mulching with traditional plastic film (T1); micro-ridge-furrow mulching with degradable film (T2); and traditional flat-cropping mulching with traditional plastic film (CK). The experimental results demonstrated that the micro-ridge-furrow mulching film planting pattern promoted the germination of rice seeds and improved the soil temperature, plant height, leaf area, dry mass, and grain yield. T2 had the highest average soil temperature (14.68–17.83 ℃ during the day; 14.4–15.74 ℃ at night), leaf area (41.85 cm^2^ plant^−1^), root dry mass (45.32 mg plant^−1^), shoot dry mass (58.46 mg plant^−1^), root–shoot ratio (0.821), and yield (8.112 t ha^−1^). In summary, the micro-ridge-furrow mulching with degradable film (T2) is recommended as an efficient planting and mulching pattern for sustainably solving environmental problems and improving grain yield in semiarid regions of China.

## Introduction

Rice (*Oryza sativa* L.) is a species of herbaceous rice, also known as Asian Cultivated Rice. Rice, wheat, and maize are the world’s three most important food crops, with nearly half of the global population dependent upon rice^1^. According to the United Nations’ Food and Agriculture Organization (FAO) statistics, there are 111 countries in the world that produce rice, and the total harvested area is 159 million ha^2^. Therefore, rice plays an extremely important role in grain production.

Due to global warming caused by greenhouse gas emissions, climate change has caused extreme weather, such as drought and extreme temperatures^1^. It was reported that in 2018, the world experienced 16 cases of drought and 26 cases of extreme temperature, which have affected food safety and fresh water supply^3,4^. Drought is one of the most complex hydroclimatic disasters because its severity is difficult to quantify; its effects include crop yield decrease or failure, famine, and ecological damage^5^. In the United States of America, between 1980 and 2020, drought caused $250 billion in damage and nearly 3000 deaths, making it the costliest and second most serious natural disaster^6^. From the autumn of 2009 to the spring of 2010, a severe drought in southwest China caused drinking water shortages affecting about 21 million people and economic losses of nearly $30 billion^7^. Experts predict that by 2050 more than 27% of the world’s major cities, with a total population of 233 million, will exhaust their current water resources^8^. Therefore, reducing greenhouse gas emissions to mitigate climate change and saving water are important ways to achieve sustainable development^3,4^.

Rice transplanting is an important conventional process for rice cultivation^9^. However, rice transplanting is not only labour-intensive but also water-intensive^10^. In addition, flooded paddy fields account for 18% of the total methane (CH_4_) emission into the atmosphere, which is a major greenhouse gas^4^. Therefore, rice transplanting no longer meets the requirements of modern agricultural development. Experts have predicted that because of global climate change, rice cultivation will be reduced by 51% in the next century, which will threaten food security and sustainable development^4^; therefore, measures must be taken to develop more sustainable rice production. Previous research has found that dry direct-seeded rice can not only save water resources, but also reduce methane emissions by 16–54% compared to rice transplanting^4^; hence, dry direct-seeded rice is an important alternative to alleviate these challenges^11,12^. Dry direct-seeded rice is directly sown in the field, convenient to implement, and does not require raising and transplanting seedlings^13^. The benefits and drawbacks of different planting patterns are shown in Table 1.

**Table 1:** Benefits and drawbacks of different planting patterns.

Planting pattern	Benefit	Drawback
Rice transplanting	Shortens the growth cycle of rice in the field, which is beneficial to the planting of the next crop Ensures the basic seedling integrity of the field and allows reasonably close planting according to the agronomic requirements	High water consumption More labour employment and high technical requirements
Dry direct-seeded rice	Less labour employment, easy to cultivate, and convenient Less water consumption	Susceptible to weather, causing a shortage of seedlings in the fields Susceptible to weed damage
Dry direct-seeded rice with film mulching	Less labour employment, easy to cultivate, and convenient Less water consumption Increases water retention and soil temperature High emergence rate and fewer weeds	Increased cost of production due to the economic input of plastic film Difficulty in disposing of the plastic film that has been used

The yield of direct-seeded rice is comparable to that of rice transplanting^14^, but dry direct-seeded rice has higher field management requirements than that of rice transplanting. This is especially true in the early stage of rice growth, which is easily affected by climate, weeds, and other factors^15,16^. In view of the climate and weed problems faced by dry direct-seeded rice cultivation, experts have researched several variations, such as improving rice varieties^17^, adjusting agronomic parameters^18,19^, and film-covering seeding^20^. Film-covering seeding has proven to be one of the most effective solutions. Dry direct-seeded rice with film mulching could improve soil temperature to promote rice seed germination, preserve moisture, and inhibit weed growth^21,22^. Moreover, soil mulching promotes soil carbon sequestration, which is an important approach to realize negative greenhouse gas emissions^23^. Meanwhile, carbon accumulation within soils can improve soil fertility and increase crop yield^24^. Li et al.^25^ reported that plastic film mulching (PM) with no flooding could enhance soil temperature, accelerate root growth, and produce similar or higher rice grain yield when compared to traditional flooding (TF) management.

The use of plastic film ensures the increase and stability of the dry direct-seeded rice yield. However, the extensive use of traditional plastic film has caused serious white pollution, which has affected the ecological environment and food safety^26–28^. Incineration is one of the most common ways to deal with traditional plastic film^29^, but the gas generated by incineration pollutes the atmosphere and accelerates climate change^30^. As one of the measures to deal with the serious white pollution, degradable materials technology has been widely used in food packaging and agricultural production^31–33^. Therefore, degradable film may be an important substitute for dry direct-seeded rice mulch in the future. However, there are few studies on degradable film in dry direct-seeded rice, and there are no relevant reports on the effect of degradable film on rice growth and yield. Still, the application of degradable film on other crops has achieved good results. Cirujeda et al.^34^ observed that degradable plastic film resulted in up to 80–100% weed control in tomato production, the same as polyethylene (PE) plastic film. The yields of degradable plastic film treatments were 72–108% of the PE plastic film treatment. Yin et al.^35^ found that degradable plastic film with a suitable degradation rate could improve soil environment, promote the growth of maize plants, and increase income.

Experts carried out research on the planting pattern of ridges and furrows with plastic film mulching to give full consideration to the role and potential for dry direct-seeded crops and improve the utilization rate of natural rainfall. The planting pattern of ridge-and-furrow mulching and rainwater harvesting is to cover the ridges and furrows with plastic film and sow seeds in the furrows. When it rains, rainwater will flow from the ridges to the furrows and penetrate the soil to moisten seeds or roots through the seed holes. The ridge-and-furrow planting pattern with plastic film mulching to collect rainwater has been widely applied to save water and improve the yield of many staple crops, such as wheat and maize, and has achieved good results^36,37^. Fan et al.^38^ reported that ridge-and-furrow film mulching (RFFM) enhanced microbial communities, soil electrical conductivity, and soil environment, and increased potato yields by 46.4–97.3% when compared with conventional flat plot (FP) without film mulching. Fan et al.^39^ found that a film fully-mulched ridge–furrow (FMRF) with a water harvesting system infused 65.7–82.7% of rainwater into the soil, doubling the soil moisture around the plant roots and enhancing the average corn yield by 14.5–22.7% when compared with conventional flat planting with mulching one-half area of soil surface (FMCF). However, whether the technology will have similar benefits on rice cultivation remains unknown, and there have been no reports on the application of this technology in dry direct-seeded rice in northern China.

In order to mitigate the effects of global climate change on rice production and the impact of traditional rice production on the climate, while at the same time addressing the environmental pollution due to traditional plastic film and make full use of natural rainfall, this study proposes a micro-ridge-furrow mulching with degradable film for dry direct-seeded rice and explores the effects of this planting pattern on soil temperature, growth of seedlings, and grain yield in a semiarid region of China. This study provides an energy-saving, efficient, and environmentally friendly planting technology for dry direct-seeded rice.

## Materials and methods

### Climate and soil characteristics

Three different field experiments were conducted in a semiarid region of China in 2019. The experimental plot is located at Wudaohezi Village, Haolibao Town, Jalaid Banner, Hinggan League, Inner Mongolia Autonomous Region, P.R. China (46°35′43″N, 123°04′36″E; 174 m in altitude), with a temperate continental monsoon climate and an annual average temperature of 4.4 ℃. The annual average precipitation is 430 mm and is mainly concentrated from June to August. The annual average frost-free period is 130 days. The soil type is meadow soil. After ploughing, the soil is finely broken and flat. The organic matter mass fraction and the pH value of the topsoil are 22.36 g/kg and 5.89, respectively.

### Plastic film performance

Two types of plastic film were used in the study: A—a traditional plastic film (Jialiming New Material Corp. Ltd., Hinggan League, China), and B—a degradable plastic film (Shanghai Hongrui Biotech, Shanghai, China). The two types of plastic film were black, 0.01 mm thick, and 1550 mm wide. A transmittance/fog tester (Manufacturer: Shanghai Shenguang Instrument & Meter Co., Ltd.; Model Number: WGT-S) was used to measure the light transmittance of the two types of plastic film. A microcomputer-controlled electronic universal material testing machine (Manufacturer: Shanghai Hengyi Precision Instrument Co., Ltd.; Model Number: Hy-0580) was used to measure the tensile strength, elongation, and elastic modulus of the films. Performance measurements of each film were repeated three times and the details are shown in Table 2.

**Table 2:** Properties of mulching films.

Film	Transmittance (%)	Tensile strength (MPa)	Elongation (%)	Elastic modulus (MPa)
A	4.50	11.90	469.28	263.27
B	6.48	9.51	278.45	148.80

### Experiment design

**Figure 1:**
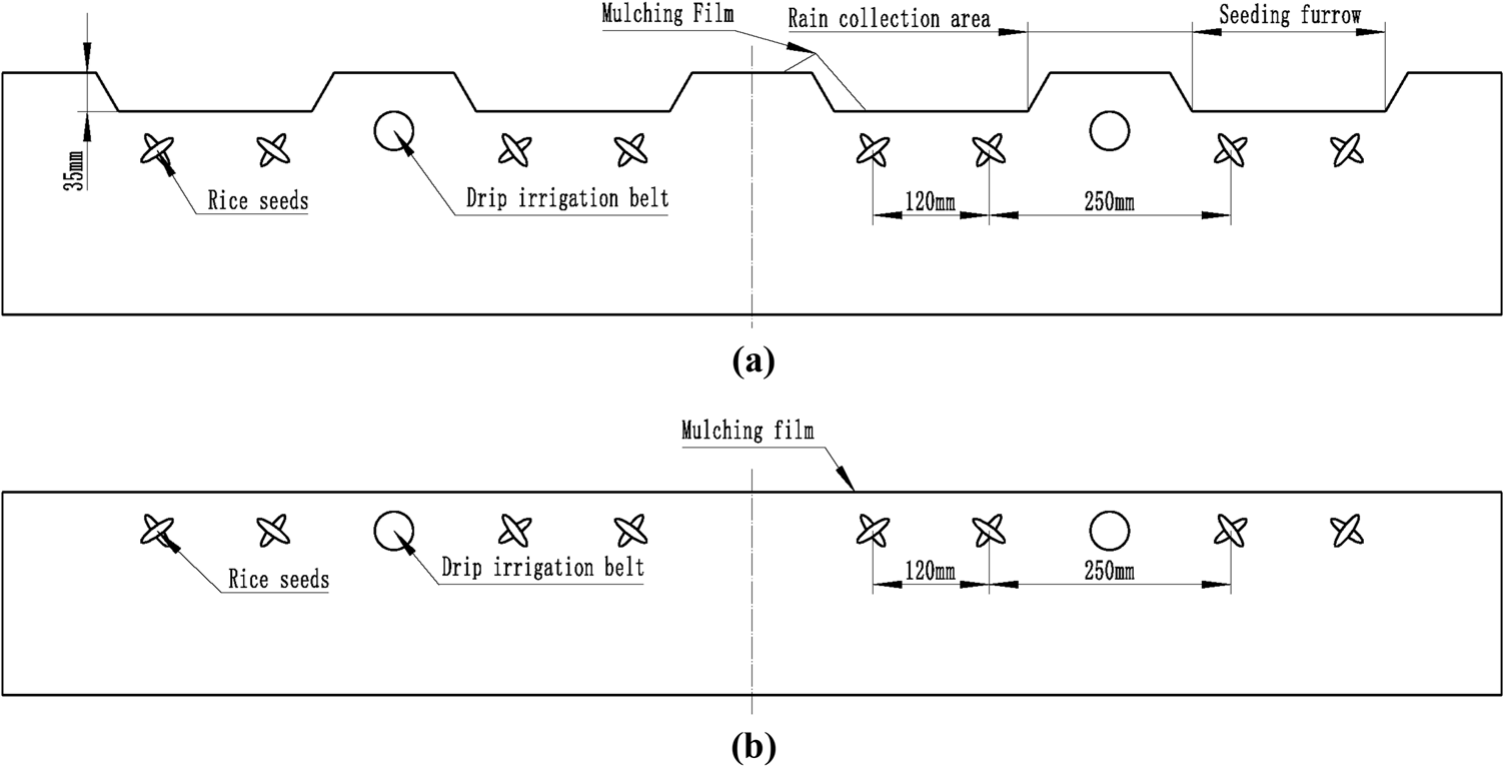
Planting pattern diagram. (**a**) Micro-ridge-furrow rainwater harvesting with mulching planting pattern; (**b**) Traditional flat-cropping planting pattern.

The experiment was two-factor and two-level. The two categoric factors were planting pattern and plastic film. The planting patterns conducted were (a) micro-ridge-furrow with mulching to collect rainwater, and (b) traditional flat-cropping, as shown in Fig. 1a,b, respectively. Plastic films A and B were used as covering materials for the two planting patterns. Three treatments were applied: T1, T2, and CK. The combination of (b) and A was utilized as the control group (CK), and the T1 and T2 treatments served as the experimental group, as shown in Table 3.

**Table 3:** Treatment of experiment.

Treatment	Planting pattern	Film
T1	(a)	A
T2	(a)	B
CK	(b)	A

An existing planter was used for the dry direct-seeded rice, sowing 8 rows at a time with an operation width of 1.5 m in the experimental plot. The operation length of a single treatment was 50 m, and each treatment was repeated 3 times. The total area of the experimental plot was approximately 1000 m^2^. All three treatments were completed by the planter for dry direct-seeded rice.

The experimental plots and fields were ploughed and base fertilizer was uniformly applied on April 10, 2019. Topdressing was applied twice in the rice growing period, on June 5 and June 20. The three treatments for field management and water use were consistent with the local field, as was the weeding. The rice variety used in the experiment was Suijing 18, which was the rice variety with the largest local planting area. Rice seeds were sown on April 29, 2019, with an average of 12 seeds per hole and a hole spacing of 120 mm. Rice in the experimental plot was harvested from September 25 to 28, 2019. Therefore, the whole experimental period lasted for more than 5 months, from April 10 to September 28, 2019.

### Sampling and measurement

Dry direct-seeded rice is susceptible to the influence of climate and weeds in the early stage of growth, but the influence is reduced after the three-leaf stage, similar to transplanting. Therefore, the experiment mainly focused on the soil temperature and the growth status of the seedlings in the early stage of rice growth and the effects of different treatments on the grain yield.

#### Soil temperature

A set of temperature sensors (Manufacturer: Sonbest Company of Shanghai; Model Number: KM3002B; Configuration: three probes) were used to collect the soil temperature of 50, 100, and 150 mm soil layers for each treatment. The temperature acquisition system collected and stored data every 30 min.

#### Germination rate

After the first water supply through a drip irrigation belt, the rice seeds met the conditions for germination. Five days later, the germination situation of the three rice seed treatments was evaluated for the first time. Subsequently, the germination information of rice seeds was collected every day until the germination rate remained constant. The germination rate of rice seeds was calculated by collecting 10 holes in each treatment at a time, and the mean value was used as the germination rate of the treatment.

1 $$ {\text{Q }} = \, \left( {{\text{n }}/{\text{ N}}} \right) \, \times { 1}00\% , $$

where Q is the germination rate, %; n is the number of buds, and N is the number of seeds.

#### Plant height and leaf area

Plant height and leaf area are important indexes of seedling growth. After the tri-foliate stage, 50 rice seedlings were selected for each treatment, and the plant height was measured using a steel ruler. An LA-S series plant image analyser (Manufacturer: Wseen Ltd., Hangzhou, China; Model Number: LA-S Series) was used to measure the leaf area of the rice plant and 10 rice seedlings were harvested for each treatment. The plant height and leaf area were averaged from the measured data.

#### Biomass

Biomass is an important index for measuring the accumulation of organic matter and nutrient composition of rice plants. In this experiment, 10 rice seedlings from each treatment were selected and excavated as a whole. After washing and drying, the rice plants were cut into two parts at the top of the root system with scissors and placed separately into drying containers. The drying containers were put into an electric thermostatic drying oven (Manufacturer: Shanghai Heheng Instrument & Equipment Co., Ltd.; Model: DHG-9050A). The temperature of the electric thermostatic drying oven was set at 105 ℃ and maintained for 30 min. The temperature of the electric thermostatic drying oven was then adjusted to 80 ℃, and the drying containers were weighed with a high-precision electronic balance (Model: Hengji Electronic Analytical Weighing Scale FA1204; Precision level: level 1; Range: 120 g; Division value: 0.1 mg) at intervals of 2 h until the weight no longer changed.

#### Grain yield

Three randomly selected points (1 m^2^) were harvested for each treatment in the experimental plot. The harvested ear of rice was shelled by hand and then weighed. The average value of the three weights was the grain yield of the treatment.

#### Degradation progress of film

In the study, the degradation process of the mulching film was recorded by visual assessment. The main purpose of collecting the degradation information of the film was to know whether the film has an impact on the growth of rice and affects the next sowing season. The degradation progress of the film was observed from sowing (on April 29, 2019) to the next sowing season (on April 15, 2020).

### Statistical analysis

Data processing and analysis were performed using Microsoft Excel and Design Expert software. Significant differences were tested using the least significant difference (LSD) method.

## Results

### Soil temperature

**Figure 2:**
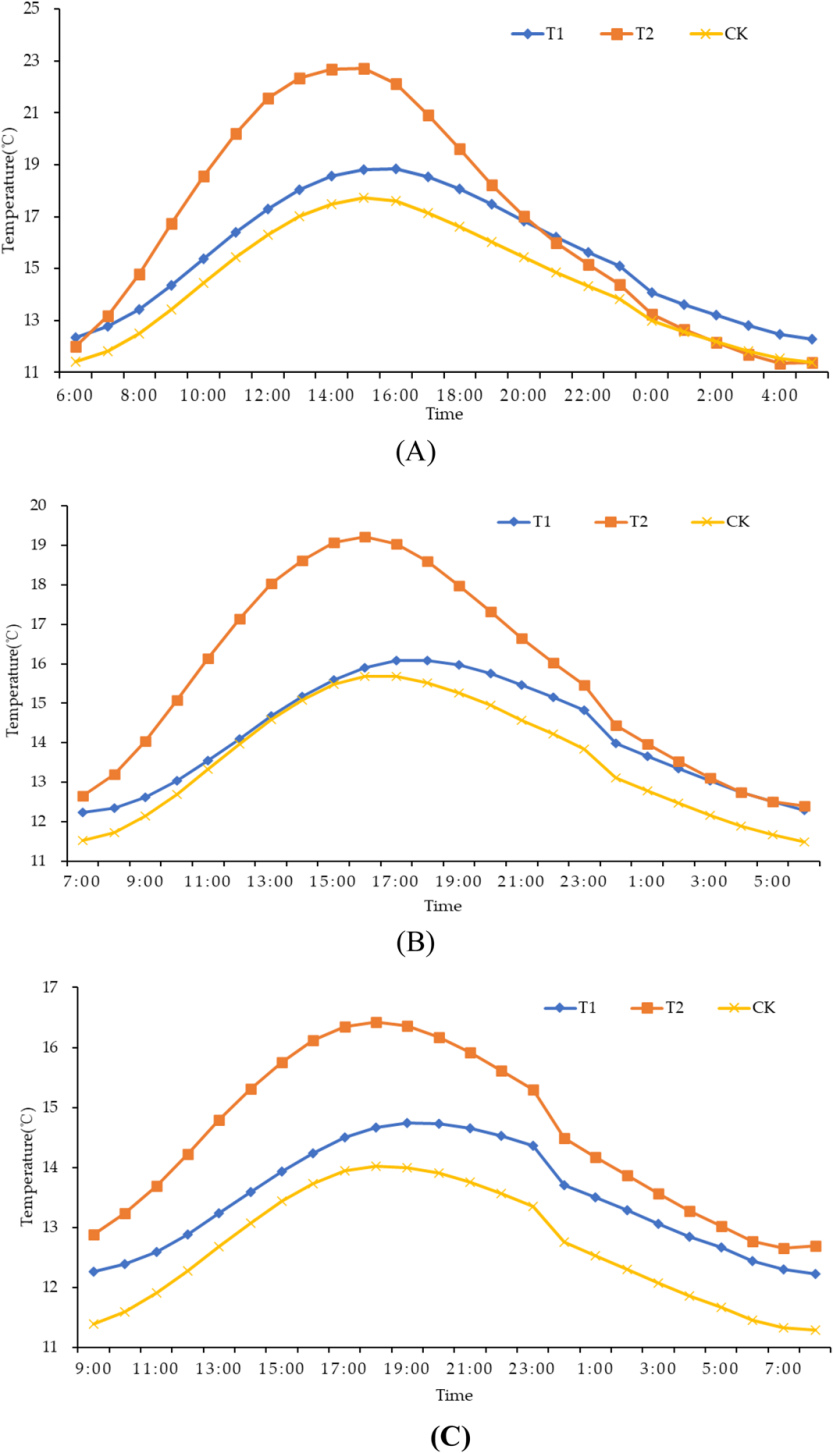
Daily soil temperature in different soil depth. (**A**) 50 mm soil depth. (**B**) 100 mm soil depth. (**C**) 150 mm soil depth.

A higher soil temperature was detected at the 50 mm depth from 6:00 to 2:00 for T1 when compared to CK. At the 50 mm depth, T1 showed a higher soil temperature from 6:00 to 21:00 but lower from 21:00 to 6:00 compared to T2. T2 showed higher soil temperature at the 50 mm depth than CK. Compared with CK, T1 and T2 showed higher soil temperatures at 100 and 150 mm depths. Compared with T2, a higher soil temperature was detected at 100 and 150 mm depths for T1, as shown in Fig. 2.

During the warming stage, T1 and T2 significantly enhanced the average soil temperature at the 50 mm depth by 8.54% and 20.8%, respectively, as compared with CK. The average soil temperature at the 100 mm depth significantly increased by 6.24% and 19.82% for T1 and T2, respectively, as compared to CK. T1 and T2 average soil temperatures at 150 mm depth significantly increased by 5.78% and 14.6%, respectively, when compared to CK, as shown in Table 4.

**Table 4:** Average soil temperature of different treatments.

Treatment	Warming stage (℃)			Insulation stage (℃)		
	50 mm	100 mm	150 mm	50 mm	100 mm	150 mm
T1	16.02b	14.47b	13.55b	15.11a	13.92b	13.41b
T2	17.83a	16.32a	14.68a	15.74a	15.27a	14.4a
CK	14.76c	13.62c	12.81c	14.16c	13.55b	12.56c

Different lowercase letters followed by the same column among the treatments means significant differences (p < 0.05) according to LSD.

During the insulation stage, the average temperature in the soil at the 50 mm depth significantly increased by 6.71% and 11.16% for T1 and T2, respectively, as compared to CK. Compared with CK, T1 and T2 significantly increased the average temperature in the soil at the 100 mm depth by 2.73% and 12.7%, respectively. As compared with CK, the average temperature in the soil at the 150 mm depth significantly increased by 6.77% and 14.64% for T1 and T2, respectively, as shown in Table 4.

### Germination rate

**Figure 3:**
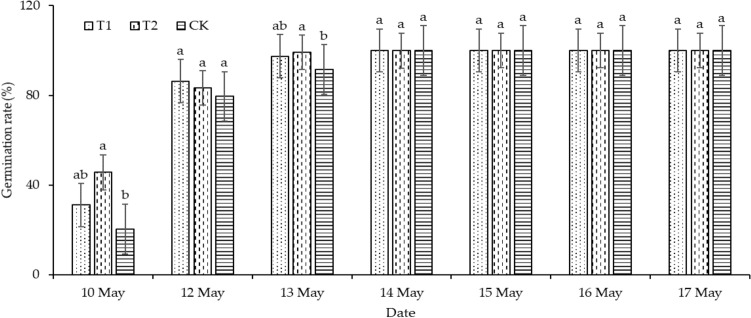
Germination rate of different treatment.

On May 10, the germination rate of T1 and T2 increased by 52.44% and 123.58%, respectively, compared with CK. The germination rate of T1 and T2 increased by 8.47% and 4.77%, respectively, on May 12, compared with CK. The germination rate of T1 and T2 as compared to CK increased by 6.39% and 8.27%, respectively, on May 13, as shown in Fig. 3.

### Seedling growth

**Figure 4:**
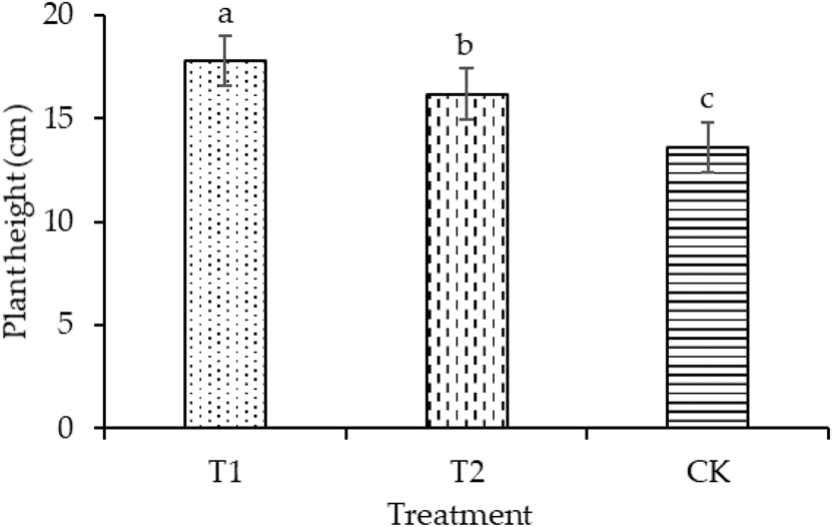
Plant height of seedling under different treatments.

**Figure 5:**
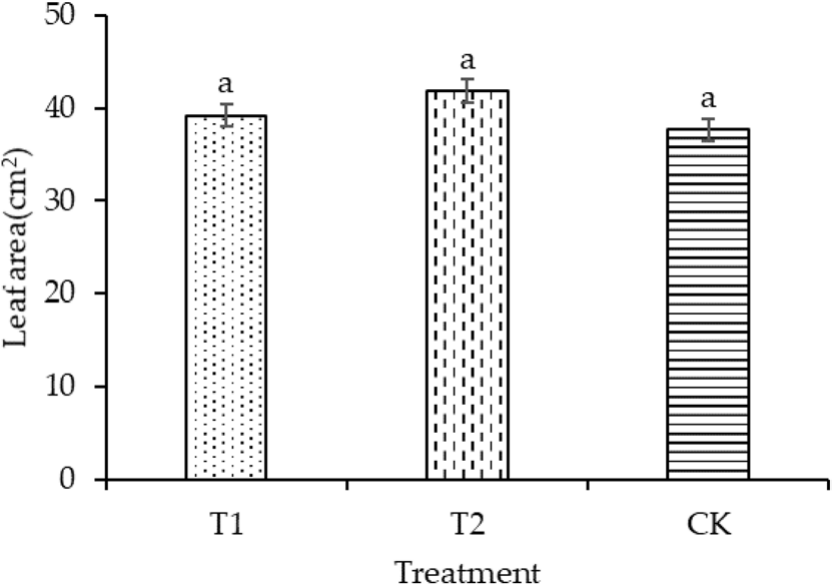
Leaf area of seedling under different treatments.

**Figure 6:**
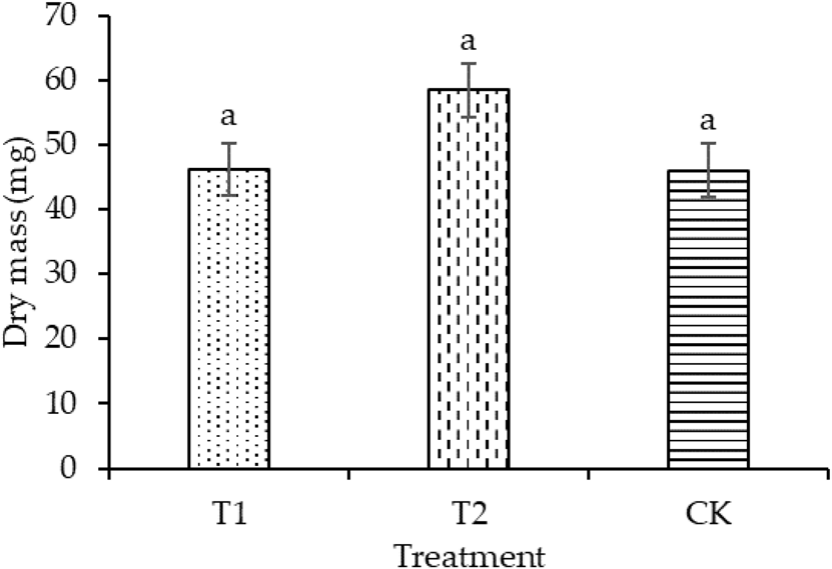
Dry mass in shoot of seedling under different treatments.

**Figure 7:**
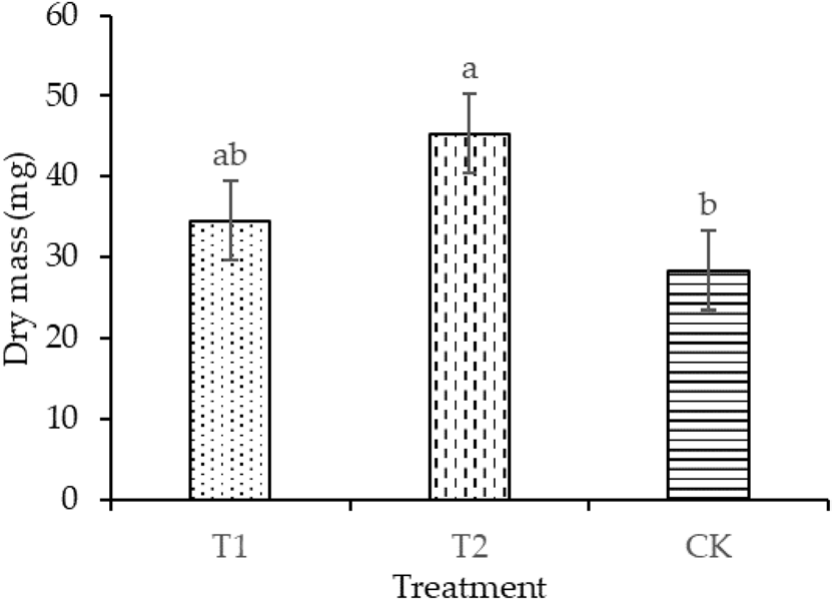
Dry mass in root of seedling under different treatments.

**Figure 8:**
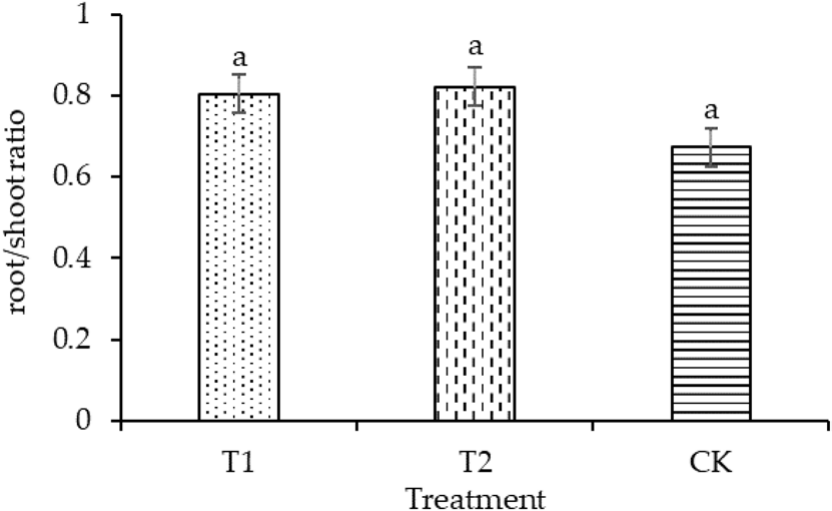
Root/shoot ratio of seedling under different treatments.

The plant height of T1 and T2 was significantly enhanced by 30.88% and 19.11%, respectively, compared with that of CK, as shown in Fig. 4. Compared with CK, the leaf areas of T1 and T2 increased by 4.14% and 11.16%, respectively, as shown in Fig. 5. T2 had the maximum dry mass in shoot (58.49 mg plant^−1^) and root (45.32 mg plant^−1^), as shown in Figs. 6 and 7, respectively. As shown in Fig. 8, T2 had the highest root–shoot ratio, with no significant difference detected among the specimens.

### Grain yield

**Figure 9:**
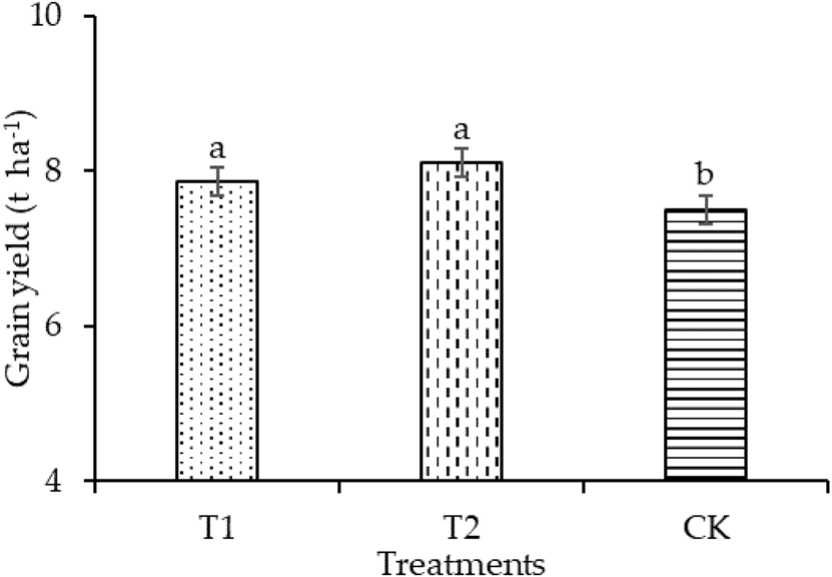
Grain yield under different treatments.

The highest grain yield was detected for T2 (8.112 t ha^−1^). Compared with CK, TI and T2 significantly enhanced grain yield by 4.79% and 8.1%, respectively, as shown in Fig. 9.

### Degradation progress of film

The induction period, cracking period, major cracking period, and fragmentation period of B occurred on July 19, July 28, September 7, and September 17, respectively. There were only a few residue films of B in the soil during tillage, which had no effect on sowing. Film B had good degradation. Film A showed no signs of degradation.

### Correlation analysis

Germination rate, as measured on May 10 was significantly related to leaf area, root dry mass, and average temperature at 50 mm soil depth during warming stage, average temperature at 150 mm soil depth during warming stage, and average temperature at 150 mm soil depth during insulation stage. Leaf area and root dry mass were highly correlated with the average temperature at 50, 100, and 150 mm soil depth during warming stage, while leaf area was extremely correlated with root dry mass. Correlation analysis results are shown in Table 5.

**Table 5:** Correlation analysis between the investigated parameters.

Index	A001	A002	A003	A004	A005	A006	A007	A008	A009	A010	A011	A012	A013	A014	A015
A002	0.4876														
A003	0.9241	0.7841													
A004	− 0.9063	− 0.0728	− 0.6760												
A005	0.5422	0.998*	0.8221	− 0.1362											
A006	0.998*	0.4341	0.8995	− 0.9301	0.4906										
A007	0.9104	0.0826	0.6832	− 1.000**	0.1459	0.9336									
A008	0.998*	0.4260	0.8955	− 0.9333	0.4827	1.000**	0.9368								
A009	0.8742	0.8501	0.9934	− 0.5870	0.8820	0.8434	0.5950	0.8385							
A010	0.9812	0.6467	0.9805	− 0.8078	0.6940	0.9679	0.8135	0.9655	0.9514						
A011	1.000*	0.4736	0.9179	− 0.9129	0.5288	0.999*	0.9169	0.999*	0.8664	0.9780					
A012	0.9924	0.3763	0.8700	− 0.9514	0.4346	0.998*	0.9544	0.999*	0.8078	0.9500	0.9942				
A013	0.999*	0.4588	0.9112	− 0.9196	0.5145	1.000*	0.9234	0.999*	0.8579	0.9744	1.000*	0.9959			
A014	0.9794	0.6540	0.9823	− 0.8021	0.7009	0.9654	0.8079	0.9630	0.9543	1.000**	0.9760	0.9470	0.9722		
A015	0.9735	0.2750	0.8123	− 0.9789	0.3358	0.9855	0.9809	0.9870	0.7401	0.9112	0.9770	0.9943	0.9805	0.9072	
A016	0.999*	0.5249	0.9398	− 0.8871	0.5780	0.9947	0.8916	0.9937	0.8944	0.9887	0.998*	0.9861	0.997*	0.9872	0.9627

* and ** significant at 0.05 and 0.01 level, respectively.

A001: germination rate on May 10; A002: germination rate on May 12; A003: germination rate on May 13; A004: germination rate on May 14; A005: plant height; A006: leaf area; V007: aboveground dry mass; A008: root dry mass; A009: root/shoot ratio; A010: grain yield; A011: average temperature at 50 mm soil depth during warming stage; A012: average temperature at 100 mm soil depth during warming stage; A013: average temperature at 150 mm soil depth during warming stage; A014: average temperature at 50 mm soil depth during insulation stage; A015: average temperature at 100 mm soil depth during insulation stage; A016: average temperature at 150 mm soil depth during insulation stage.

## Discussion

Rapid and stable seed germination and seedling establishment are important for rice growth and yield formation. Appropriate soil temperature is an important condition for rapid germination and crop growth. Previous studies have shown that film mulching can effectively increase soil temperature^40^, and ridge-furrow planting with film mulching increased soil temperature even more^41^. Mo et al.^42^ reported that alternating small and large ridges with full film mulching could significantly elevate soil temperature in linseed (*Linum usitatissimum* L.) planting. Gu et al.^43^ found that film-mulched continuous ridge-furrow planting improved soil temperature for a winter oilseed rape field in Northwest China. Mo et al.^44^ observed that a ridge-furrow mulching system with transparent PE film and a ridge-furrow mulching system with black PE film significantly increased topsoil temperature by 1.3 and 0.3 ℃, respectively, when compared with a ridge-furrow system without mulching. Zhang et al.^45^ reported that a ridge-furrow mulching system with plastic film increased soil temperature by 1.0 to 2.5 °C during the day and 1.6 to 3.4 °C at night, compared with flat planting without mulching. Our results align with the findings from previous studies. In this study, the micro-ridge-furrow with film mulching increased soil temperature from 6.71 to 20.8%, 2.73 to 19.82%, and 5.78 to 14.64% at 50, 100, and 150 mm soil depths, respectively, compared to the control treatment. The influence of a micro-ridge-furrow with film mulching on soil temperature decreased with increasing soil depth. Compared with T1, T2 soil temperature increased significantly by 11.3%, 12.79%, and 8.34% at 50, 100, and 150 mm soil depths, respectively. The higher daytime temperature in T2 compared to T1 was due to the high transmittance of degradable plastic film compared with traditional plastic film, as shown in Table 1, which promoted the soil absorption of solar energy. In the present study, the beneficial combination of soil moisture and temperature under a micro-ridge-furrow planting pattern with film mulching improved the germination rate from 4.77 to 123.58% compared to CK. This is consistent with the previous studies by Li et al.^46,47^. Overall, compared with a traditional flat-cropping planting pattern, a micro-ridge-furrow pattern with film mulching could significantly increase the soil temperature and promote the germination of rice seeds. Meanwhile, the overall effect on soil temperature was better for degradable film than traditional plastic film.

In the present study, the growth indicators of rice seedlings in planting pattern (a) were higher compared with those in planting pattern (b), as shown in Figs. 4, 5, 6, 7, 8. The plant heights for T1 and T2 were significantly higher than that of CK, and the plant heights for T1 and T2 were also significantly different from each other. Temperature and water are the main factors influencing the early growth of rice seedlings. A micro-ridge-furrow planting pattern with film mulching provided better soil hydrothermal conditions than a traditional flat-cropping planting pattern, promoting the growth of plants. Different covering materials affected the thermal insulation and water retention for a micro-ridge-furrow planting pattern with film mulching. T1 and T2 had the same water source, but it is assumed that the water retention ability of the degradable film was weaker than that of traditional plastic film, resulting in the plant height of T2 being greater than that of T1. No significant difference was observed in the leaf area, dry mass in root, dry mass in shoot, and root–shoot ratio among the three treatments. However, T1 and T2 were still higher than CK in these categories. Our research was consistent with previous studies. Li et al.^48^ reported that ridge–furrow plastic film mulch significantly increased plant height, relative to conventional flat planting. Zheng et al.^49^ found that a plastic-mulched ridge plus bare furrow (RP) and plastic-mulched ridge plus straw-mulched furrow (RPFS) enhanced plant height by 6.1% and 8.0% and enhanced stem diameter by 11.2% and by 14.5%, respectively, compared with flat cultivation with non-mulching (NM). Compared with NM, the leaf area index (LAI) under different mulching types was not significantly different at the early growth stage. RPFS and RP enhanced LAI by 23.6% and 13.4%, respectively. Zhao et al.^50^ observed that the plant height, LAI, and dry biomass of full mulching on double ridges and furrows (DRFFM) and RFFM were significantly higher than traditional-flat planting without mulching. Ren et al.^51^ reported that a plastic film mulching ridge-and-furrow rainfall collecting system significantly increased root parameters such as root length, root volume, and root dry weight, compared with the conventional flat practice. Dong et al.^52^ found that the crops with ridging and film cover could have increased crop growth rate, and biomass could be accumulated rapidly. This study demonstrated the same trends as the above studies. In general, micro-ridge-furrows with film mulching improved the soil conditions and promoted the growth rate of rice seedlings. Different covering materials largely influenced the effect of the micro-ridge-furrow planting pattern with film mulching.

Figure 9 shows that a micro-ridge-furrow with film mulching significantly increased the grain yield from 4.79 to 8.1% compared with CK. There was no significant difference in the grain yield between T1 and T2. A micro-ridge-furrow with film mulching raised the soil temperature to promote the germination of rice seeds and increased the growth rate of rice seedlings to promote the formation of biomass. Biomass accumulation is an important factor affecting yield. These results agreed with previous reports. Dong et al.^52^ reported that the growth of production by the ridge-furrow with film mulching was in part due to an increase in the allocation of biomass to the grains. Tian et al.^53^ ranked the positive effects on the tuber yield increase of potatoes as best for the ridge with plastic mulching, followed by the bare-earth ridge, and finally flat planting. Zhang et al.^54^ reported that ridge-furrow construction combined with a plastic film mulching system significantly increased maize grain yield from 38.0 to 59.6% compared with FP. Li et al.^55^ observed that ridge-and-furrow tillage with a mulching system significantly enhanced maize yields when compared with conventional flat tillage without mulching. They studied plastic-film mulched ridges (PFR) with plastic film (PF), biodegradable film (BR), and straw mulching (MS) on furrows and found that all of these also significantly increased the maize yield. Compared to CK, the average yield of PF + BF, PF + PF, and PF + MS were significantly enhanced, by 42.1%, 41.1%, and 39.3%, respectively. Gu et al.^56^ found that planting and mulching patterns had a significant effect on winter oilseed rape yield. Flat planting with film mulching (M1), ridge-furrow planting with film mulching on both ridges and furrows (M2), and ridge-furrow planting with film mulching on continuous ridges (M3) obtained higher yields than flat planting without mulching during the three crop seasons, and M3 had the highest yield. In summary, micro-ridge-furrows with film mulching improved the soil environment and provided better conditions for the germination of rice seeds and growth of rice seedlings, which increased the rice yield.

After ploughing, the bulk density, organic matter mass fraction, and pH value of the topsoil were 1.4 g/cm^3^, 22.36 g/kg, and 5.89, respectively. Soil carbon cycle is an important manifestation of the soil internal biochemical process^57^. Soil mulching promotes the accumulation of soil carbon; however, long-term continuous tracking is required to determine its change process and mechanism^57^. The field experiments conducted by Liu et al.^58^ and Wang et al.^59^ for more than 5 years showed that furrow and ridge planting with film mulching had no significant effect on soil organic carbon and were consistent with the conclusions of the field experiments conducted by Li^57^ for 7 years. However, through field experiments over 17 years, Li^60^ observed that furrow and ridge planting with film mulching increased the soil organic carbon content compared with that without mulch, which presented some differences to the above conclusions. Therefore, the influence of furrow and ridge planting with film mulching on the soil characteristics is a long-term and gradual accumulation process, whereas the short-term influence is not significant. Therefore, in the study, field experiments were conducted to investigate the effects of the micro-ridge-furrow mulching degradable film on rice growth and yield of dry direct-seeded rice; however, the soil characteristics will be continuously tracked and monitored in the future.

## Conclusions

In this study, it was observed that a micro-ridge-furrow with film mulching cultivation increased soil temperature, promoted seedling growth, and achieved higher grain yield. T2 demonstrated the best performance, increasing soil temperature, germination rate, plant height, leaf area, root dry mass, shoot dry mass, root–shoot ratio, and grain yield by 11.16–20.8%, 4.77–123.58%, 19.12%, 11.16%, 59.75%, 26.84%, 21.95%, and 8.1%, respectively, compared with CK. Therefore, the micro-ridge-furrow mulching with degradable film is recommended as an alternative to traditional dry direct-seeded rice planting pattern.

The results from the planting pattern and technique are encouraging, but the size and time of the experiment were limited. Therefore, in future studies, the experimental area should be expanded, and the experimental time should be extended. Additionally, the degradation rate of degradable plastic film should be further studied to examine the suitability of degradable plastic film.
